# Metabolism reprogramming signature associated with stromal cells abundance in tumor microenvironment improve prognostic risk classification for gastric cancer

**DOI:** 10.1186/s12876-022-02451-2

**Published:** 2022-07-30

**Authors:** Junyu Huo, Jing Guan, Yankun Li

**Affiliations:** 1 The Affiliated Hospital of Qingdao University, No. 59 Haier Road, Qingdao, 266003 China; 2 Department of General Surgery, Qilu Hospital (Qingdao), Cheeloo College of Medicine, Shandong University, 758 Hefei Road, Qingdao, 266035 Shandong China; 3 Department of Critical Care Medicine, Qilu Hospital (Qingdao), Cheeloo College of Medicine, Shandong University, 758 Hefei Road, Qingdao, 266035 Shandong China

**Keywords:** Gastric cancer, Metabolic, Stromal cells, Prognostic, Signature, Tumor microenvironment

## Abstract

**Background:**

Stromal cells play an important role in the process of tumor progression, but the relationship between stromal cells and metabolic reprogramming is not very clear in gastric cancer (GC).

**Methods:**

Metabolism-related genes associated with stromal cells were identified in The Cancer Genome Atlas (TCGA) and GSE84437 datasets, and the two datasets with 804 GC patients were integrated into a training cohort to establish the prognostic signature. Univariate Cox regression analysis was used to screen for prognosis-related genes. A risk score was constructed by LASSO regression analysis combined with multivariate Cox regression analysis. The patients were classified into groups with high and low risk according to the median value. Two independent cohorts, GSE62254 (n = 300) and GSE15459 (n = 191), were used to externally verify the risk score performance. The CIBERSORT method was applied to quantify the immune cell infiltration of all included samples.

**Results:**

A risk score consisting of 24 metabolic genes showed good performance in predicting the overall survival (OS) of GC patients in both the training (TCGA and GSE84437) and testing cohorts (GSE62254 and GSE15459). As the risk score increased, the patients’ risk of death increased. The risk score was an independent prognostic indicator in both the training and testing cohorts suggested by the univariate and multivariate Cox regression analyses. The patients were clustered into four subtypes according to the quantification of 22 kinds of immune cell infiltration (ICI). The proportion of ICI Cluster C with the best prognosis in the low-risk group was approximately twice as high as that in the high-risk group, and the risk score of ICI Cluster C was significantly lower than that of the other three subtypes.

**Conclusion:**

Our study proposed the first scheme for prognostic risk classification of GC from the perspective of tumor stromal cells and metabolic reprogramming, which may contribute to the development of therapeutic strategies for GC.

**Supplementary Information:**

The online version contains supplementary material available at 10.1186/s12876-022-02451-2.

## Background

Tumor tissue has a complex microenvironment. The oxygen content, lactic acid concentration and nutrient supply in different regions of tumor tissue are different, but tumor cells can adapt to adversity and maintain rapid growth. This adaptation is achieved by changing the energy metabolism of tumor cells, which is called energy metabolism reprogramming [1].

In recent years, studies have found that the occurrence and development of tumors are not only related to changes in the gene structure and phenotype of tumor cells but also closely related to the tumor microenvironment [2]. Tumor stromal cells account for approximately 50% of the total number of cells in tumor tissue [3] and play an important role in tumor metabolism, growth, metastasis, immune escape and chemotherapy resistance [4–6]. On one hand, stromal cells are induced by tumor cells to undergo metabolic reprogramming, increase aerobic glycolysis, produce a large number of metabolites, generate nutrients, provide energy and nutrition for tumors, and maintain tumor biosynthesis; on the other hand, stromal cells can regulate the metabolic reprogramming of tumor cells to cope with the nutritional pressure of the tumor microenvironment [7, 8]. At present, the metabolic reprogramming of stromal cells and the mechanism of promoting tumor metastasis and drug resistance are still unclear. However, blocking the interaction between stromal cells, tumors and the immune microenvironment provides a new research direction for tumor therapy.

The incidence of digestive system tumors is the highest in the world, approximately 50–60%, and the phenomenon of metabolic reprogramming of tumor cells is widespread [9]. Gastric cancer (GC) accounts for the initiation of all kinds of digestive tract tumors and ranks third among all tumors [10]. In-depth study of the characteristics and molecular mechanism of tumor metabolic reprogramming and the development of effective means to regulate metabolic reprogramming may provide new ideas for the comprehensive treatment of GC.

In this study, we attempted to explore the relationship between metabolic reprogramming and stromal cells, especially their effect on the prognosis of GC and discuss the possible mechanism to provide new directions for the treatment of GC.

## Materials and methods

### Data collection

The gene expression data and clinical information were obtained from The Cancer Genome Atlas (https://portal.gdc.cancer.gov/) and the Gene Expression Omnibus (GEO) database (https://www.ncbi.nlm.nih.gov/geo/). We obeyed the access rules of the TCGA and GEO databases during the process of data acquisition. The data utilized in this work were acquired from public databases, and approval from the local ethics committee was not needed. The clinical data of the included samples are shown in Table 1. The workflow of the study is displayed in Additional file 1: Fig. S1.

**Table 1:** The clinical data of the included samples

	TCGA	GSE84437	GSE62254	GSE15459
Survival status				
Alive	226	224	148	96
Dead	145	209	152	95
Gender				
Female	133	137	100	67
Male	238	294	195	124
Age				
≤ 65	163	282	172	87
> 65	205	149	123	104
Stage				
I			30	31
II			94	29
III			95	72
IV			76	59
Lauren classification				
Diffuse			134	75
Intestinal			144	98
Mixed			17	18
Stage T				
T1–2	96	49		
T3	167	92		
T4	100	290		
Stage N				
N0	108	80		
N1	97	187		
N2	74	132		
N3	74	32		
Lymphovascular				
Yes			171	
No			62	

### Identification of metabolism-related genes associated with the StromalScore

The ESTIMATE (Estimation of STromal and Immune cells in MAlignant Tumor tissues using Expression data) algorithm generates the stromal score by analyzing the specific gene expression characteristics of stromal cells to quantify the stromal components in the tumor tissues [11, 12]. We calculated the stromal score with the ESTIMATE algorithm to estimate the stromal cell abundance of the TCGA and GSE84437 datasets. Patients were assigned to high- and low-stromal score groups according to the optimal cutoff determined by the R package “survminer” in the two independent cohorts. The metabolism-related genes that encoded all the known metabolic enzymes and human transporters were obtained from a previously published paper [13]. The differentially expressed metabolism-related genes (DEMRGs) between the high- and low-StromalScore groups were identified by the R package “limma” in the two independent datasets. A false discovery rate (FDR) < 0.001 was considered statistically significant.

### Metabolic gene cluster analysis

The TCGA cohort with 371 GC patients and the GSE84437 with 433 GC patients were merged into a training cohort (n = 804). The batch effects between the TCGA and GSE84437 datasets were removed by the combat function in the R package “sva”. Cluster analysis was performed on the intersection DEMRGs of the two datasets based on the R package “consensusclusterplus”.

### Development and validation of a metabolic-related gene prognostic signature for GC

First, univariate Cox regression analysis was applied to screen DEMRGs associated with prognosis (*p* value < 0.05 was considered significant). Next, the least absolute shrinkage and selection operator (LASSO) algorithm with penalty parameter tuning performed via tenfold cross‐validation was used to remove overfitting between the prognosis-related genes. Finally, genes with nonzero regression coefficients were included in multivariate Cox regression analyses. The formula of a risk score was formed by the multivariate Cox regression coefficient of each mRNA multiplied by each normalized mRNA expression level [14]. According to the median risk score, patients were divided into the low-risk group and the high-risk group. The R package "glmnet" was used to perform LASSO regression analysis. The R packages "survminer" and "survivalROC" were utilized to generate the Kaplan–Meier survival curve and the time-dependent ROC curve for assessing the predictive ability of the risk score. Two independent cohorts, GSE62254 (n = 300) and GSE15459 (n = 191), were used to externally verify the risk score performance.

### The quantification of immune cell infiltration (ICI)

The CIBERSORT method was applied to quantify the immune cell infiltration of all included samples, and samples with *p* < 0.05 were clustered into different subtypes according to the results of ICI with the R package “consensusclusterplus”.

## Results

### GC patients with a higher StromalScore had a poor prognosis

**Fig. 1:**
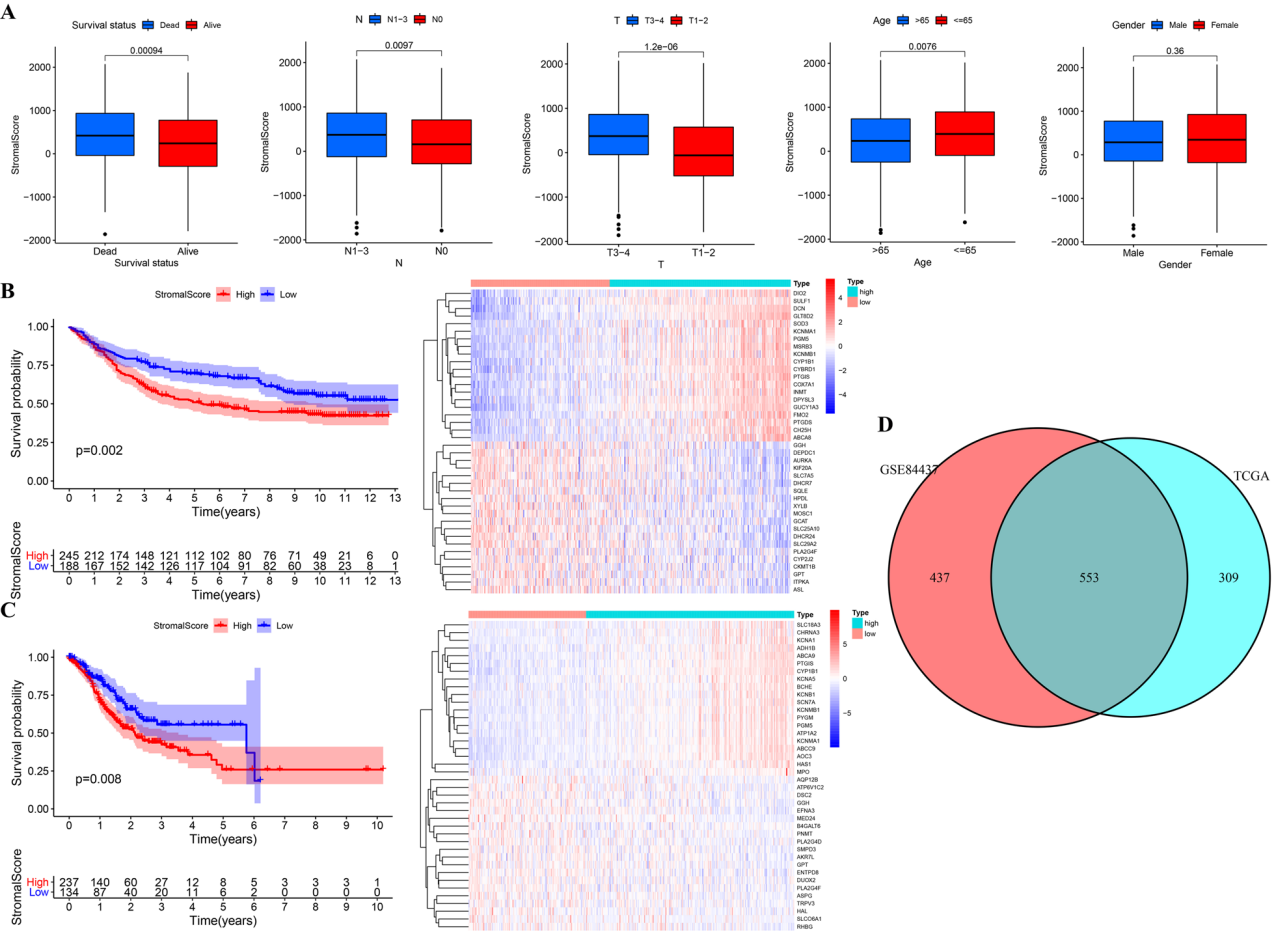
Identification of metabolism-related genes associated with the StromalScore. **A** Association between the StromalScore and clinicopathological features. **B** Kaplan–Meier survival analysis based on the StromalScore and OS in the TCGA cohort and the heatmap of DEMRGs between the high and low StromalScores in the TCGA cohort. **C** Kaplan–Meier survival analysis regarding the StromalScore and OS in the GSE84437 cohort and the heatmap of DEMRGs between the high and low StromalScores in the GSE84437 cohort. **D** Venn plot of intersection genes

In terms of clinicopathological features, GC patients who were younger (age no more than 65 years old), had died, or had advanced stage disease (T3–4 and N1–3) exhibited elevated stromal scores (Fig. 1A). The high stromal score group had significantly lower overall survival (OS) than the low stromal score group in the TCGA and GSE84437 cohorts (Fig. 1B, C). We obtained 553 intersection genes by identifying the DEMRGs between the low- and high stromal score groups of the two independent cohorts (Fig. 1D).

### The prognosis of GC was associated with the metabolic gene cluster

**Fig. 2:**
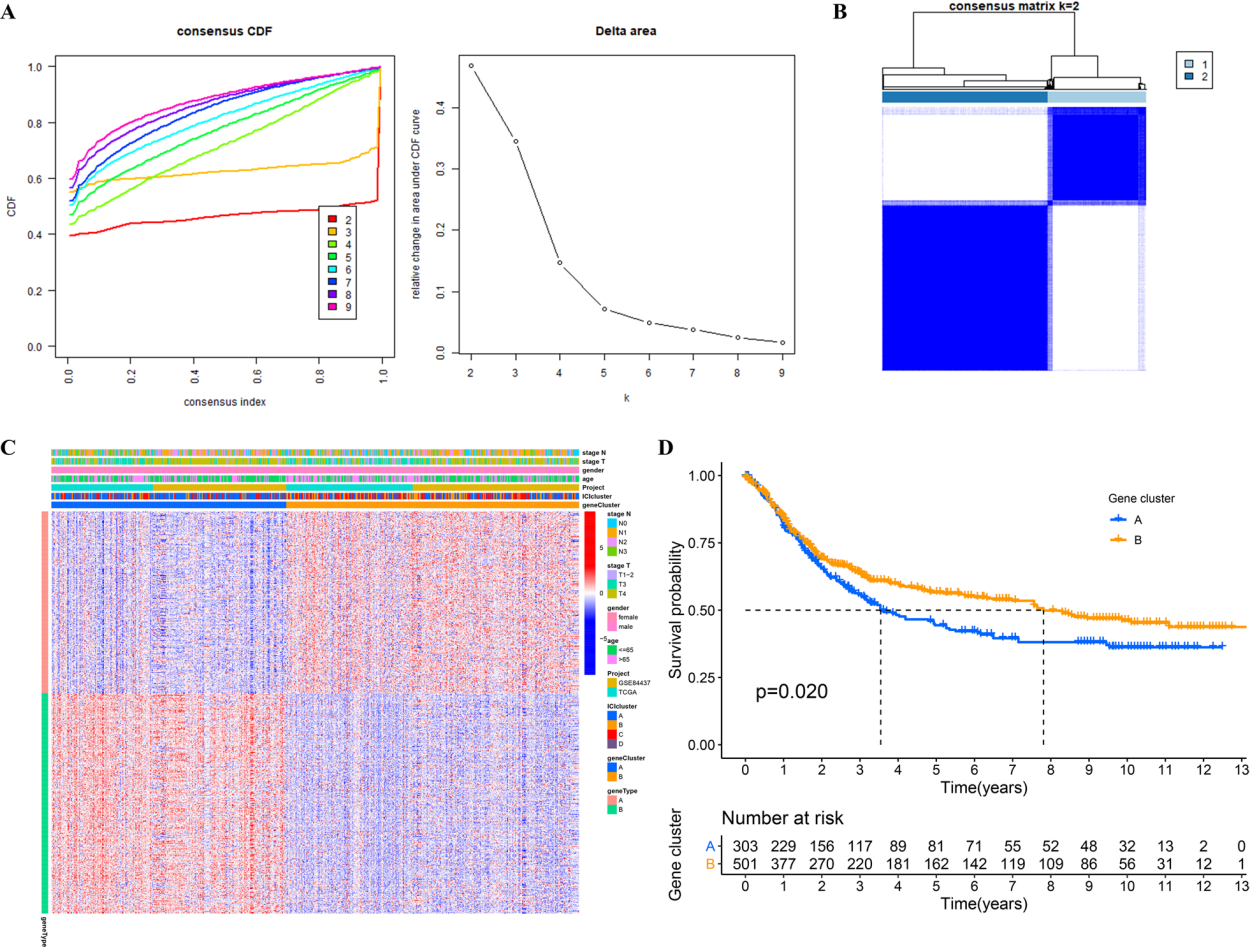
Metabolic gene cluster analysis. **A** CDF plot. **B** Clustering heatmap. **C** Heatmap of metabolic gene clusters. **D** Kaplan–Meier survival analysis regarding metabolic gene clusters and OS in the training cohort

Using the K-means clustering algorithm, the CDF plot showed that the optimal number of clusters was 2 (Fig. 2A) when the consensus matrix (k) varied from 2 to 9. At this point, the intragroup showed the highest correlation, and the intergroup showed the lowest correlation (Fig. 2B). Therefore, the training cohort was clustered into two subtypes based on the 553 intersecting metabolic genes (Fig. 2C). The OS of subtype A was obviously worse than that of subtype B (Fig. 2D), indicating that these 553 intersection metabolic genes had a significant correlation with the OS of GC.

### A 24-metabolic gene prognostic signature established in the training cohort

**Fig. 3:**
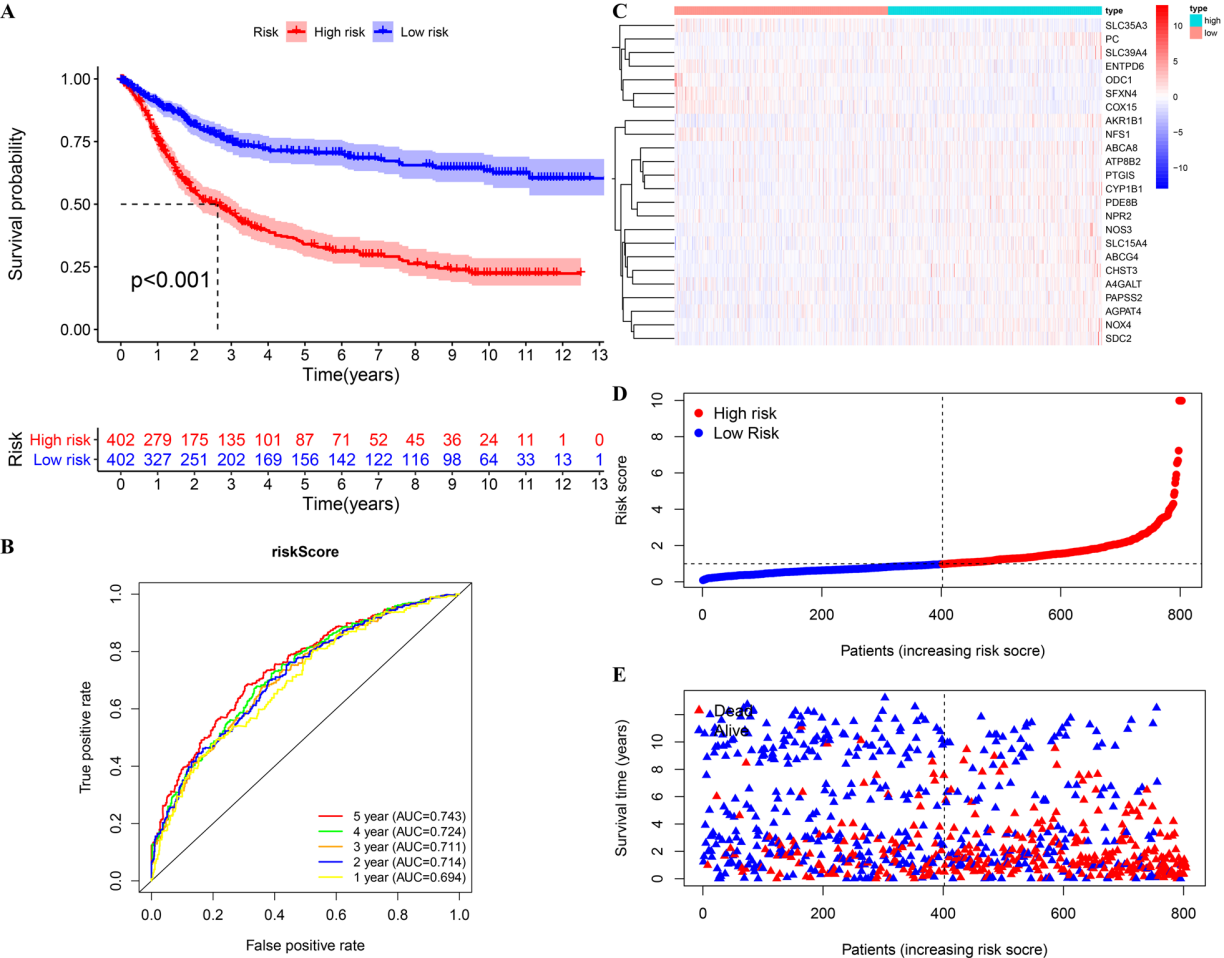
Prognostic assessment of the risk score in the training cohort. **A** Kaplan–Meier survival analysis regarding risk score and OS in the training cohort. **B** Time-dependent ROC analysis of the risk score predicting the OS of patients in the training cohort. **C**–**E** Heatmap, risk score distribution and survival status of patients in the training cohort

Using a *p* value < 0.05 as a screening criterion, 179 genes were predicted to be prognostic candidate biomarkers by univariate Cox regression analysis (Additional file 3: Table S1). Thirty-seven genes with nonzero LASSO regression coefficients were retained for multivariate Cox regression analysis (Additional file 2: Fig S2A). A model was chosen based on the Akaike information criterion (AIC) [15] using a stepwise algorithm, and 24 genes from the formula were used to calculate the risk score (Additional file 2: Fig S2B, Table 2). A total of 402 patients with a risk score greater than the median value (0.995) were assigned to the high-risk group, which showed significantly reduced overall survival (OS) compared to the 402 patients with a risk score less than the median value (0.995) (Fig. 3A). The area under the curve (AUC) values for the risk score predicting OS at 1, 3 and 5 years were 0.694, 0.711 and 0.743, respectively (Fig. 3B). As the risk score increased, the patients’ risk of death increased (Fig. 3C–E).

**Table 2:** The gene name and coef

Gene name	Coef
A4GALT	− 0.14487
ABCA8	0.189365
ABCG4	0.405526
AGPAT4	− 0.20345
AKR1B1	0.037458
ATP8B2	− 0.10627
CHST3	0.060912
COX15	− 0.10802
CYP1B1	0.047021
ENTPD6	− 0.0271
NFS1	− 0.15496
NOS3	0.086105
NOX4	0.213579
NPR2	− 0.14221
ODC1	− 0.01242
PAPSS2	0.039518
PC	0.171004
PDE8B	0.158436
PTGIS	− 0.06516
SDC2	0.02765
SFXN4	− 0.06339
SLC15A4	0.1562
SLC35A3	− 0.06357
SLC39A4	0.017992

### The risk score was an independent prognostic indicator for GC

**Fig. 4:**
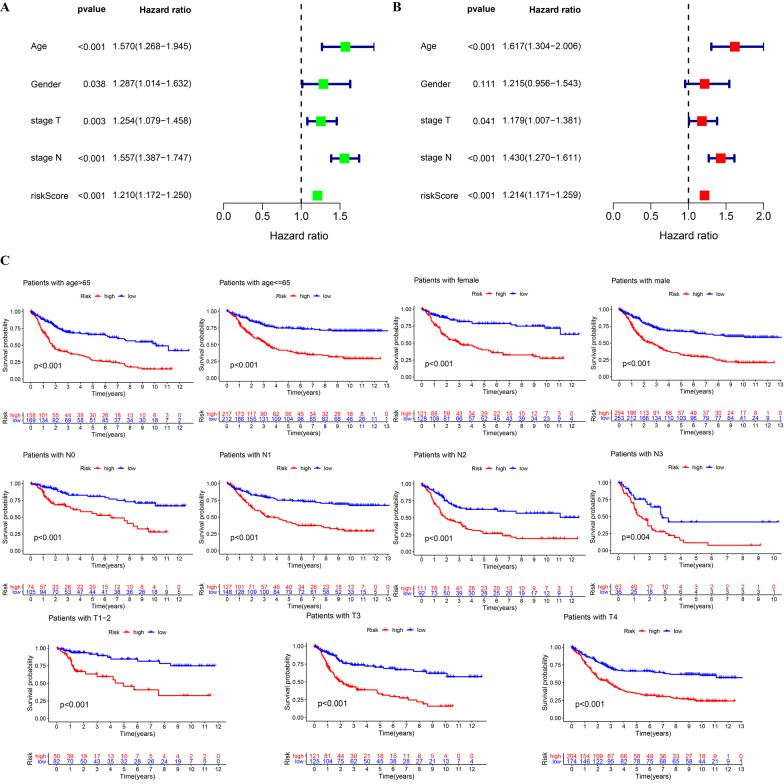
Clinical subgroup validation of the prognostic risk score. **A** The forest plot of the univariate Cox analysis. **B** The forest plot of the multivariate Cox analysis. **C** Clinical subgroup survival analysis

By analyzing 756 cases with complete clinical data in the training cohort, we found that risk score, T stage and N stage were independent prognostic indicators in univariate and multivariate Cox regression analysis (Fig. 4A, B). Next, the patients were divided into 11 subgroups for verification according to age, sex, T stage and N stage. We found that the OS of the high-risk group was significantly lower than that of the low-risk group in each subgroup (Fig. 4C), indicating that the risk score has universal applicability to the prognosis classification of GC patients.

### Internal validation of the prognostic signature in the TCGA and GSE84437 cohorts

**Fig. 5:**
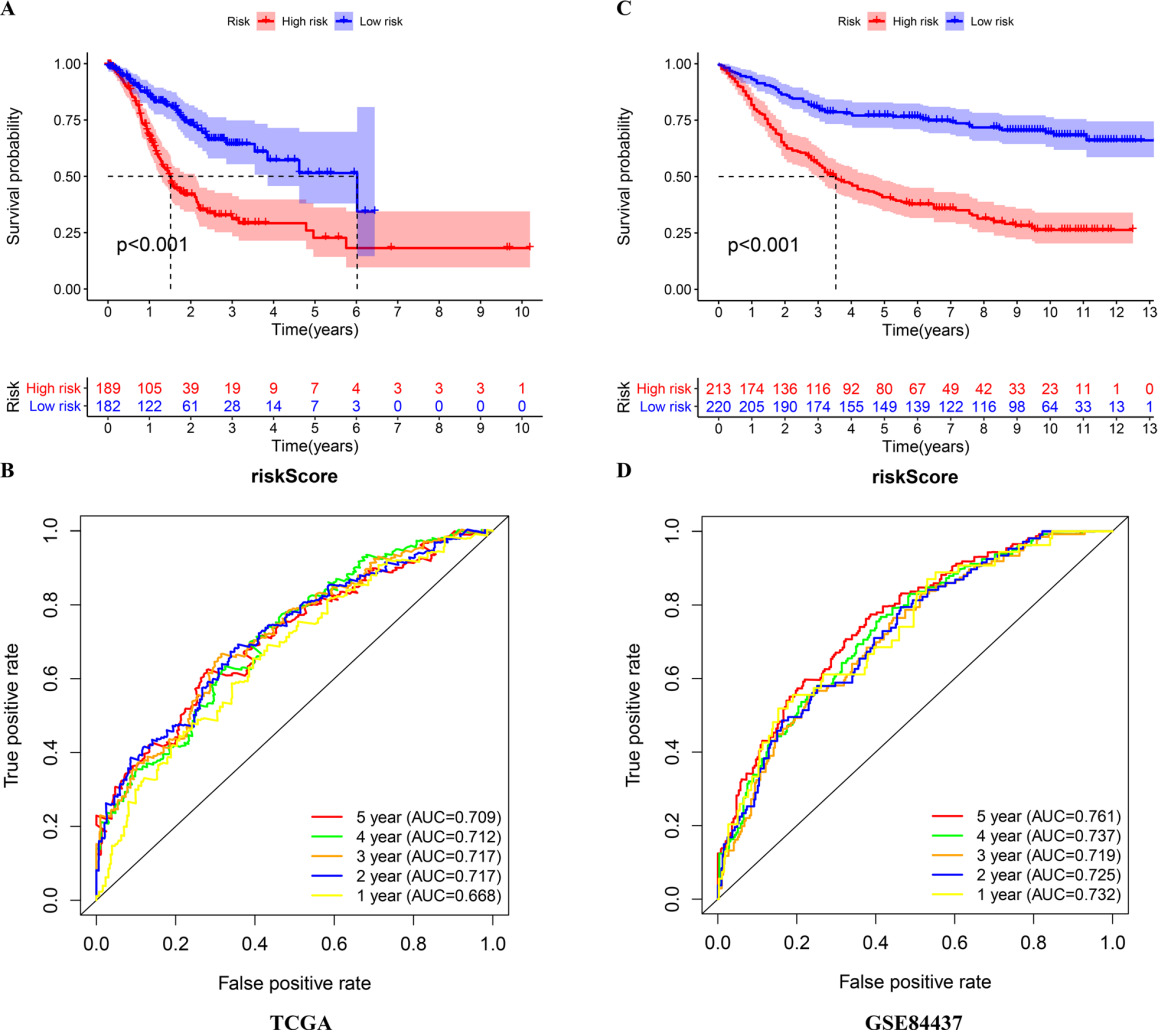
Internal validation of the risk score in the TCGA and GSE84437 cohorts. **A**, **B** Kaplan–Meier survival analysis and time-dependent ROC analysis of the signature for predicting the OS of patients in the TCGA cohort. **C**, **D** Kaplan–Meier survival analysis and time-dependent ROC analysis of the signature predicting the OS of patients in the GSE84437 cohort

In the TCGA and GSE84437 cohorts, the high-risk patients showed significantly worse OS than the low-risk patients (Fig. 5A, C). The AUC values of the risk score predicting the OS of GC patients in the TCGA cohort at 1, 3 and 5 years were 0.668, 0.717 and 0.709, respectively (Fig. 5B). The AUC values of the risk score predicting the OS of GC patients in the GSE84437 cohort at 1, 3 and 5 years were 0.732, 0.719 and 0.761, respectively (Fig. 5D).

### External validation of the prognostic signature in the GSE15459 and GSE62254 cohorts

**Fig. 6:**
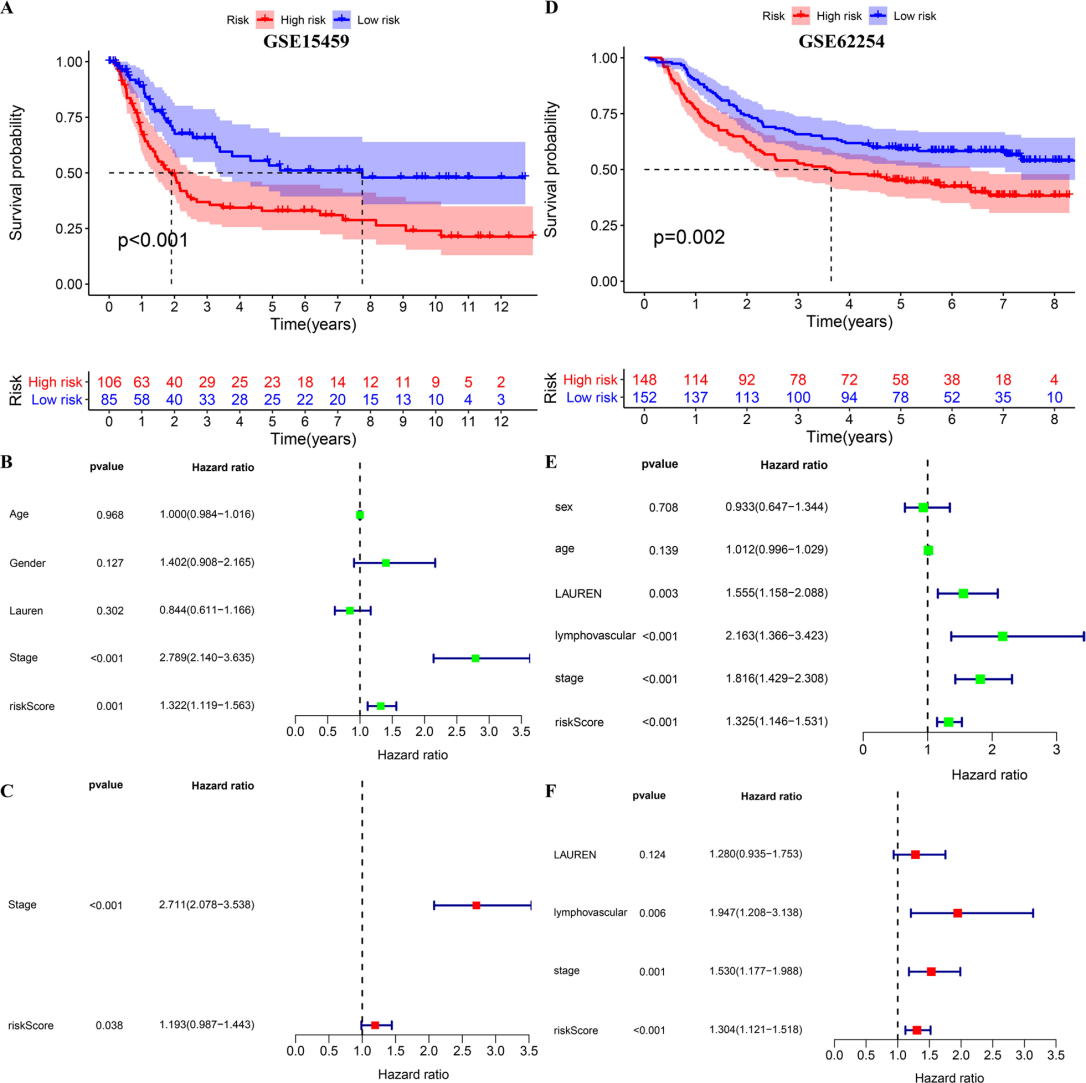
External validation of the risk score in the GSE15459 and GSE62254 cohorts. **A**–**C** Kaplan–Meier survival analysis, the forest plot of the univariate Cox analysis and the multivariate Cox analysis in the GSE15459 cohort. **D**–**F** Kaplan–Meier survival analysis, the forest plot of the univariate Cox analysis and the multivariate Cox analysis in the GSE62254 cohort

Compared to the high-risk group, the low-risk group had better clinical outcomes in the GSE15459 and GSE62254 cohorts (Fig. 6A, D). As suggested by the univariate and multivariate Cox regression analyses, the risk score independently predicted the OS of GC patients (Fig. 6B, C, E, F).

### Exploration of the relationship between immune cell infiltration characterization and the prognostic signature

**Fig. 7:**
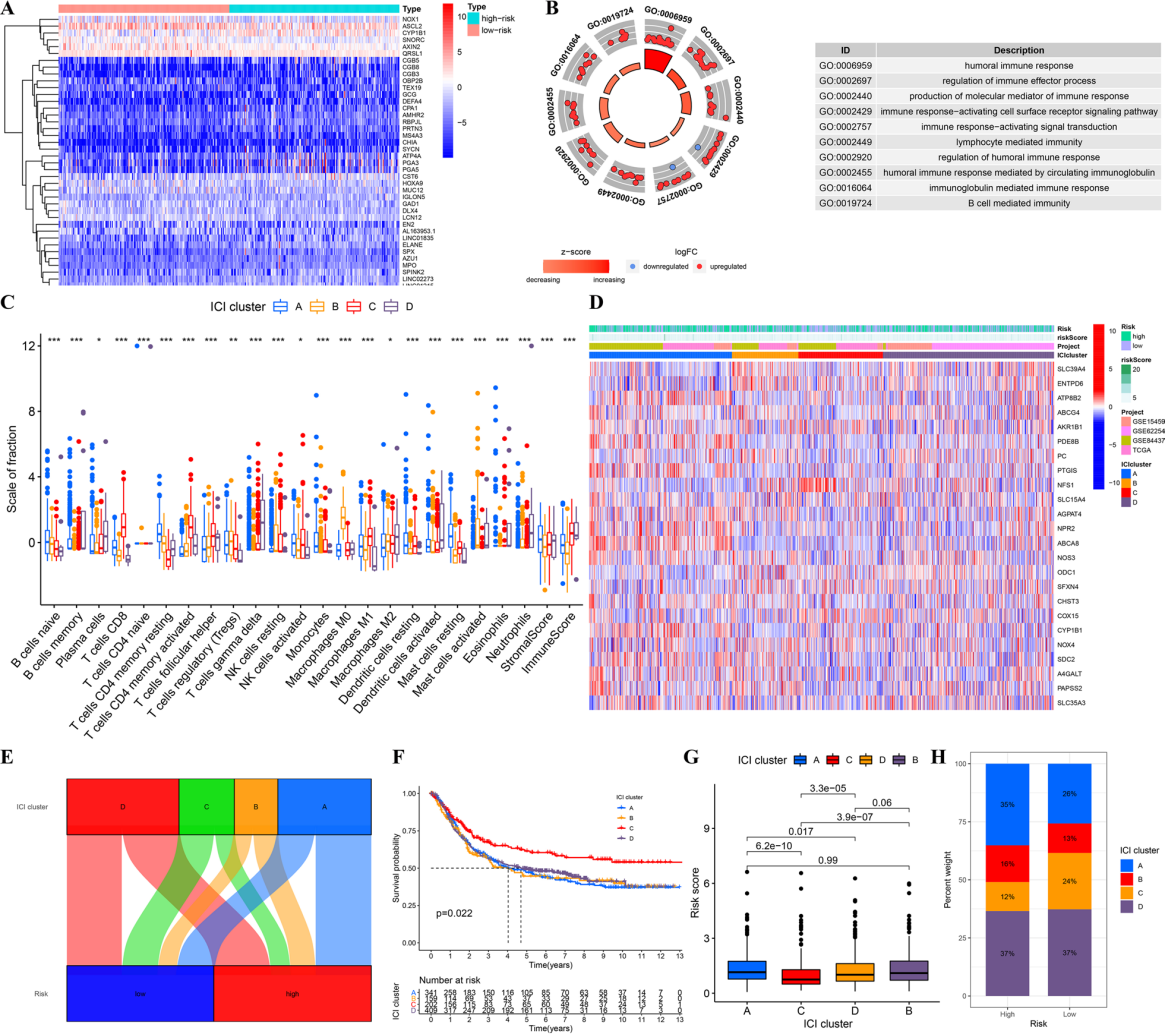
Relationship between the risk score and immune cell infiltration (ICI) characterization. **A** Heatmap of DEGs between high- and low-risk groups. **B** GO term annotation of DEGs. **C** Boxplot of the four ICI clusters. **D** Distribution of four ICI clusters in high- and low-risk groups. **E** Distribution of ICI clusters in different risk groups **F** Kaplan–Meier survival analysis of different ICI clusters. **G** Boxplot of the risk score difference among the four ICI clusters. **H** Bar plot of proportions of the four ICI clusters in high- and low-risk groups

To determine the underlying mechanism of the prognostic signature, we identified differentially expressed genes (DEGs) between the high- and low-risk groups (Fig. 7A). GO term annotation showed that these DEGs were mainly involved in immune response-related biological processes (Fig. 7B). Thus, we hypothesized that the metabolic reprogramming signature may reshape the immune microenvironment of GC and attempted to investigate the association between the signature and immune cell infiltration (ICI). Patients were clustered into four subtypes according to the quantification of 22 kinds of ICIs. In terms of immune infiltration characteristics, the infiltration proportion of naïve B cells, resting memory CD4 T cells, and resting mast cells in ICI-A was the highest; ICI-B was accompanied by a large number of regulatory T cells (Tregs) and infiltrating M0 macrophages. The proportion of M1 macrophages and CD8 T cells in ICI-C was significantly higher than that in the other three subtypes. ICI-D had the highest infiltration of plasma cells and the lowest infiltration of Tregs (Fig. 7C). Interestingly, the expression levels of signature-related genes varied among different ICI subtypes (Fig. 7D), indicating that the metabolic pattern of different ICI subtypes also changed. Four ICI subtypes were distributed in both the high-risk and low-risk groups (Fig. 7E). Among them, the prognosis of ICI-C was significantly better than that of the other three subtypes (Fig. 7F). Furthermore, the risk score of ICI-C was significantly lower than that of the other three subtypes (Fig. 7G). It is worth mentioning that the ICI-C subtype accounted for 24% of the low-risk group, which was twice as high as that in the high-risk group (Fig. 7H).

## Discussion

Tumors are composed of tumor cells and their microenvironment. Under the environmental selection pressure of the microenvironment and genetic factors, tumors evolve internally. At the same time, within the restriction of the genotype, the metabolic characteristics of tumors change adaptively. This process is called metabolic reprogramming [1, 16]. Numerous studies have shown that the presence of stromal cells induces tumor metabolic reprogramming, promotes tumor adaptation to nutritional deficiency, and maintains the occurrence and development of tumors [7, 8, 17].

Due to in-depth study of tumor mechanisms and energy metabolism characteristics, a large number of antitumor drugs have been applied in the clinic. In the research and application of targeted tumor therapy, how to obtain long-term and effective therapeutic effects or find a better therapeutic strategy that can specifically kill tumor cells is a difficult problem in the clinic [18, 19]. The study of the tumor cell microenvironment and energy metabolism will be conducive to the targeted therapy of antitumor drugs. From the perspective of energy metabolism, we can explore the mechanism of tumor formation and formulate specific tumor treatment methods from the perspective of energy blocking to inhibit tumor proliferation [20, 21].

Although the overall survival rate of gastric cancer (GC) has significantly improved with the diversification of treatment methods, as the malignant digestive tract tumor has the highest incidence in the world, the precise treatment of GC still faces some obstacles. A precise prognostic evaluation system is undoubtedly the key to determining the success or failure of GC treatment strategies. The tumor microenvironment (TME) has become a new target for investigating tumor pathogenesis and curbing tumor progression. However, in the field of GC, related research is not sufficient, especially with regard to the role of stromal cells in the TME.

In this paper, we focused on tumor stromal cells and metabolic reprogramming as clues. We first found that the abundance of stromal cells had a significant effect on the OS of GC, and the expression pattern of GC metabolic genes changed with the abundance of stromal cells. Based on cluster analysis of 553 overlapping metabolic genes from two independent datasets (TCGA, n = 371; GSE84437, n = 433), we found that the expression patterns of metabolic genes related to the abundance of tumor stromal cells had a significant impact on the clinical outcome of GC. As an independent prognostic indicator for GC verified internally and externally, the 24-gene signature identified by univariate Cox regression analysis, lasso regression analysis and multivariate Cox regression analysis showed good performance in prognostic risk stratification of GC. By quantifying the infiltration of immune cells, we found that differences in prognosis between the high- and low-risk groups may be related to immune cell infiltration characterization, and further confirmed that there is a complex interaction between metabolic reprogramming and the immune microenvironment of GC. At present, only a few gene functions in this signature have been clarified during the process of GC progression. For example, NOX4 regulates the proliferation and apoptosis of GC cells through the generation of ROS and subsequent activation of GLI1 signaling [22]. The high expression of PTGIS could promote the infiltration of TAMs and Treg cells in the GC TME and lead to poor prognosis [23]. As there are few studies on the above molecules in GC, their specific biological effects need to be verified by large sample, multicenter trials in the future. However, the gradual deepening of the understanding of these new biomarkers will effectively promote the development of individualized and precise treatment for GC.

Using TCGA as a reference cohort, we compared the predictive value of the signature with previously published GC metabolic prognostic signatures [24, 25]. Survival curves based on Luo’s metabolism signature [24] showed that that the median survival time of the high-risk group was 2 years and that of the low-risk group was approximately 4 years, while survival curves based on our signature revealed that the median survival time of the high-risk group was significantly less than 2 years, and that of the low-risk group was more than 6 years. Obviously, our signature has better distinction in the prognosis of GC. The time-dependent ROC curves showed that the AUC values for Yu’s seven-gene metabolic signature predicting OS at 3 and 5 years were 0.694 and 0.674, respectively [25], while those for our signature was 0.717 and 0.709, respectively. Therefore, compared with the results of previous studies, our signature improved the risk stratification of GC prognosis based on metabolic genes.

By analyzing high-throughput sequencing data from a public database, this study provides new insights into the prognostic assessment of GC from the perspective of tumor stromal cells and metabolic reprogramming. The results of internal and external validation showed that the prognostic model has great potential for clinical transformation. However, as a retrospective study, further prospective experiments and clinical trials are urgently needed to verify the prognostic value of these metabolic genes.

## Discussion

Our study proposed a scheme for prognostic risk classification of gastric cancer from the perspective of tumor stromal cells and metabolic reprogramming for the first time, which may contribute to the development of therapeutic strategies for gastric cancer.

## Supplementary Information

**Additional file 1: Fig. S1.** Workflow chart.

**Additional file 2: Fig. S2.** The building process of the risk score (**A**) LASSO regression analysis (**B**) multivariate Cox regression analysis.

**Additional file 3: Table S1.** 179 prognostic related genes identified by univariate Cox regression analysis.

## Data Availability

The datasets analysed for this study were obtained from The Cancer Genome Atlas (TCGA, https://portal.gdc.cancer.gov/) and Gene Expression Omnibus (GEO, https://www.ncbi.nlm.nih.gov/geo/).

## Abbreviations

GC: Gastric cancer
TCGA: The Cancer Genome Atlas
GEO: Gene Expression Ominibus
TME: Tumor microenvironment
ESTIMATE: Estimation of STromal and Immune cells in MAlignant Tumor tissues using Expression data
DEMRGs: Differential expressed metabolic-related genes
LASSO: Least absolute shrinkage and selection operator
ROC: Receiver operating characteristic
AUC: Area under curve
OS: Overall survival
ICI: Immune cell infiltration
