# Supplementary figures and images for: Metabolism reprogramming signature associated with stromal cells abundance in tumor microenvironment improve prognostic risk classification for gastric cancer
Source: BMC Gastroenterol. 2022 Jul 30;22:364. doi: 10.1186/s12876-022-02451-2 (PMC9338655; doi:10.1186/s12876-022-02451-2)

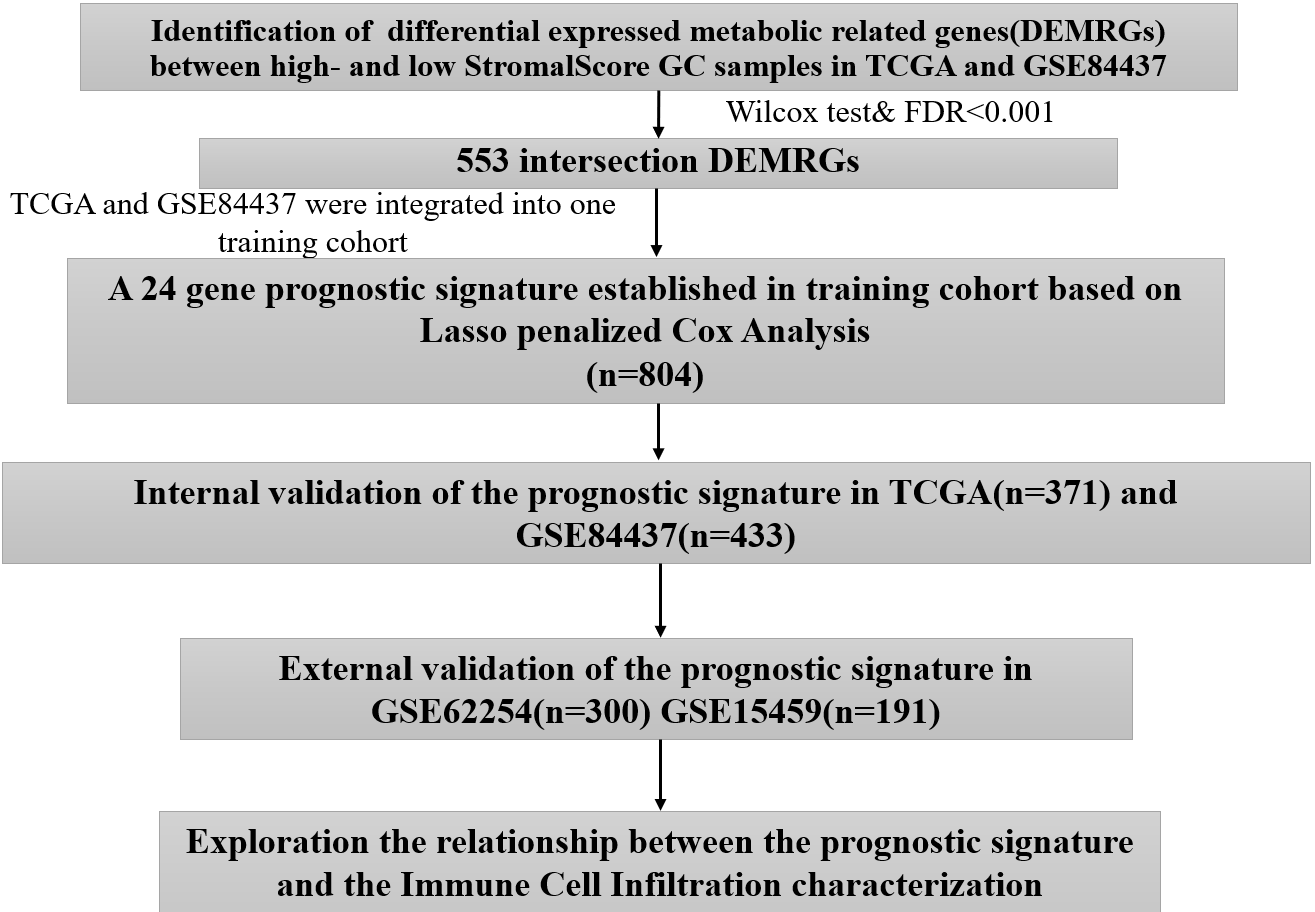

Supplement: Supplementary file 1 — Additional file 1: Fig. S1. Workflow chart. [file 12876_2022_2451_MOESM1_ESM.png]

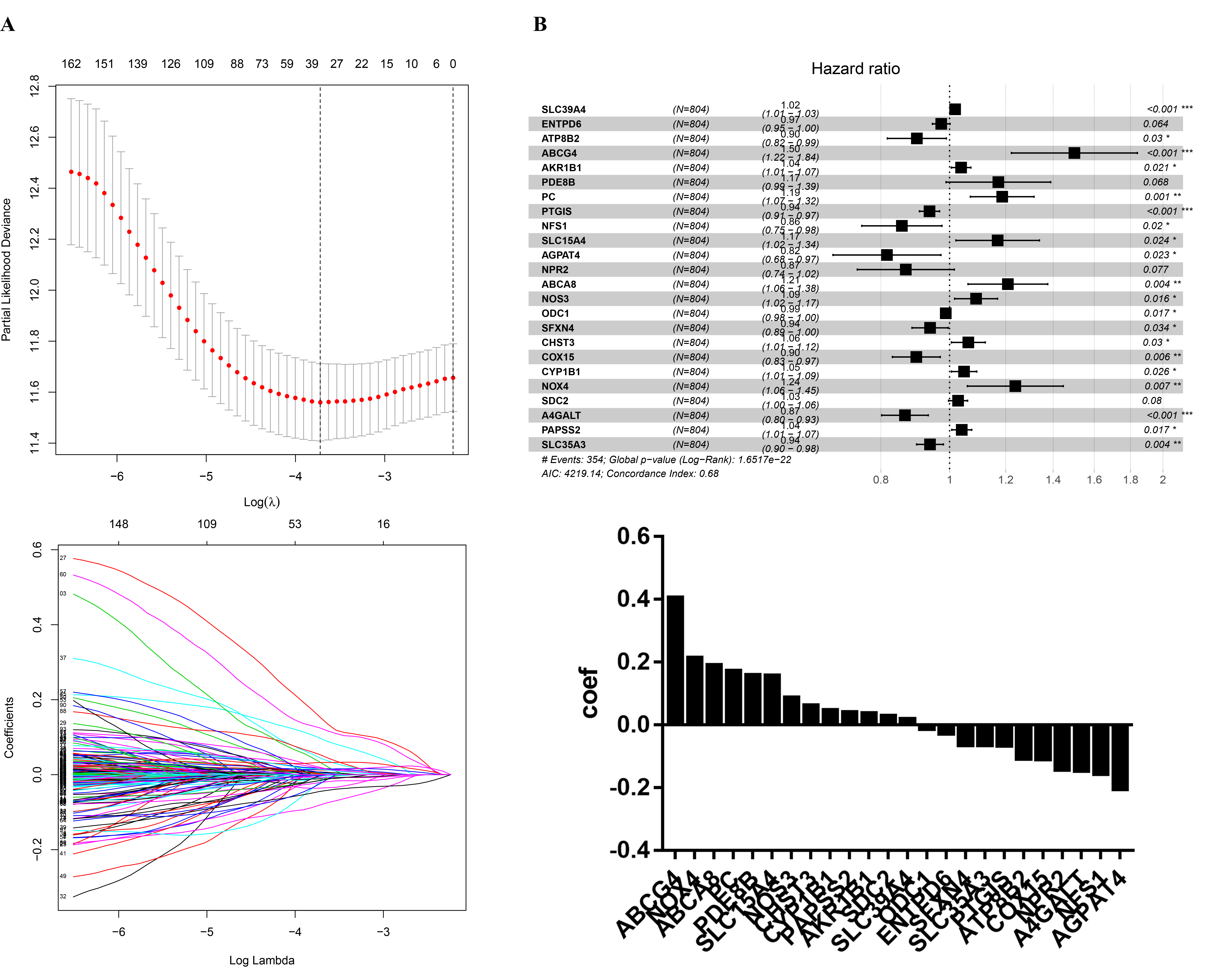

Supplement: Supplementary file 2 — Additional file 2: Fig. S2. The building process of the risk score (A) LASSO regression analysis (B) multivariate Cox regression analysis. [file 12876_2022_2451_MOESM2_ESM.png]
